# Postoperative pain treatment with erector spinae plane block and pectoralis nerve blocks in patients undergoing mitral/tricuspid valve repair — a randomized controlled trial

**DOI:** 10.1186/s12871-020-00961-8

**Published:** 2020-02-27

**Authors:** Bogusław Gawęda, Michał Borys, Bartłomiej Belina, Janusz Bąk, Miroslaw Czuczwar, Bogumiła Wołoszczuk-Gębicka, Maciej Kolowca, Kazimierz Widenka

**Affiliations:** 1 Division of Cardiovascular Surgery, St. Jadwiga Provincial Clinical Hospital, ul. Lwowska 60, 35-301 Rzeszów, Poland; 2 Second Department of Anesthesia and Intensive Care, Medical University of Lublin, ul. Staszica 16, 20-081 Lublin, Poland; 3 Anesthesiology and Intensive Care Department with the Center for Acute Poisoning, St. Jadwiga Provincial Clinical Hospital, ul. Lwowska 60, 35-301 Rzeszów, Poland

**Keywords:** Erector spinae plane (ESP) block, Pectoralis nerve (PECS) blocks, Patient-controlled analgesia (PCA), Visual analog scale (VAS)

## Abstract

**Background:**

Effective postoperative pain control remains a challenge for patients undergoing cardiac surgery. Novel regional blocks may improve pain management for such patients and can shorten their length of stay in the hospital.

To compare postoperative pain intensity in patients undergoing cardiac surgery with either erector spinae plane (ESP) block or combined ESP and pectoralis nerve (PECS) blocks.

**Methods:**

This was a prospective, randomized, controlled, double-blinded study done in a tertiary hospital. Thirty patients undergoing mitral/tricuspid valve repair via mini-thoracotomy were included. Patients were randomly allocated to one of two groups: ESP or PECS + ESP group (1:1 randomization). Patients in both groups received a single-shot, ultrasound-guided ESP block. Participants in PECS + ESP group received additional PECS blocks. Each patient had to be extubated within 2 h from the end of the surgery. Pain was treated via a patient-controlled analgesia (PCA) pump. The primary outcome was the total oxycodone consumption via PCA during the first postoperative day. The secondary outcomes included pain intensity measured on the visual analog scale (VAS), patient satisfaction, Prince Henry Hospital Pain Score (PHHPS), and spirometry.

**Results:**

Patients in the PECS + ESP group used significantly less oxycodone than those in the ESP group: median 12 [interquartile range (IQR): 6–16] mg vs. 20 [IQR: 18–29] mg (*p* = 0.0004). Moreover, pain intensity was significantly lower in the PECS + ESP group at each of the five measurements during the first postoperative day. Patients in the PECS + ESP group were more satisfied with pain management. No difference was noticed between both groups in PHHPS and spirometry.

**Conclusions:**

The addition of PECS blocks to ESP reduced consumption of oxycodone via PCA, reduced pain intensity on the VAS, and increased patient satisfaction with pain management in patients undergoing mitral/tricuspid valve repair via mini-thoracotomy.

**Trial registration:**

The study was registered on the 19th July 2018 (first posted) on the ClinicalTrials.gov identifier: NCT03592485.

## Background

Postoperative pain remains a primary challenge in patients undergoing thoracotomy [1]. Poorly managed postoperative pain is associated with an increased number of postoperative complications, including prolonged mechanical ventilation and pulmonary infections [2, 3]. Well-established pain management is an essential aspect of the Enhanced Recovery After Surgery (ERAS) protocol [4]. Recently, we have attempted to institute the ERAS protocol for cardiac surgery procedures performed in our department. Thus, an effective and safe analgesic technique was needed, which was compatible with the ERAS concept.

Among many regional anesthesia techniques for patients undergoing cardiac surgery, thoracic epidural analgesia (TEA) is associated with reduced incidences of cardiovascular events and infections, lower cost, and shortened length of hospital stay [5–7]. Thoracic paravertebral block (PVB) exhibits similar effectiveness to that of TEA for analgesia after cardiothoracic surgery [8, 9]. Other regional anesthesia techniques are not well-established in cardiothoracic surgery [10]. Novel fascial blocks, including the erector spinae plane (ESP) block and pectoralis nerve (PECS) block, have been recently proposed as effective methods of pain management for patients undergoing cardiac surgery [11, 12].

Our previous, prospective, cohort study demonstrated that the ESP block combined with low-dose intravenous oxycodone was an effective analgesic technique for patients who had undergone mitral or/and tricuspid valve repair via right mini-thoracotomy [13]. In that study, all patients could be weaned from mechanical ventilation within 2 h postoperatively and were transferred to the general ward on the second postoperative day. However, an abrupt reduction in pain intensity was observed at the 24th postoperative hour; this was clearly associated with the removal of chest drains. We hypothesized that an additional regional block, covering the area of the anterior part of the chest wall, might improve postoperative pain management [14, 15].

The objective of this study was to compare postoperative pain intensity in patients undergoing cardiac surgery with either ESP block or combined ESP and PECS blocks by assessing oxycodone consumption during the first operative day (primary objective), as well as by comparing patients’ subjective pain intensity by using the visual-analogue scale (VAS, secondary objective).

## Methods

This was a randomized, controlled, double-blind trial conducted in a tertiary cardiac surgery department. Before patient recruitment, the study protocol was approved by the Bioethics Committee of the Medical University of Lublin, Lublin, Poland (permit number KE-0254/127/2018), and registered at ClinicalTrials.gov (NCT03592485). Written informed consent was obtained from each patient, and the study was conducted in accordance with the tenets of the Declaration of Helsinki for medical research involving human subjects.

### Participants

The inclusion criteria were as follows: patients who (1) required mitral and/or tricuspid valve repair; (2) underwent surgery via right mini-thoracotomy approach; (3) were more than 18 years of age; and (4) were less than 80 years of age. The exclusion criteria included: (1) coagulopathy, defined as known bleeding disorder; (2) allergy to local anaesthetics; (3) depression, which could significantly influence pain perception; (4) epilepsy; (5) antidepressant or epileptic drug treatment; (6) chronic usage of analgesic drugs; (7) addiction to alcohol or recreational drugs. Data from patients who required endotracheal intubation and respiratory support for > 2 h from the end of surgery were also excluded from the analysis.

### Intervention

Patients were randomly allocated to one of two groups (1:1 ratio, parallel randomization) via computer-generated randomization conducted by a team member who was not involved in the surgery or patient assessment. The same team member prepared opaque envelopes in which the intervention type was concealed. These envelopes were opened a few minutes before attempting the regional block. Patients were randomly assigned to the ESP or PECS + ESP group.

**Fig. 1:**
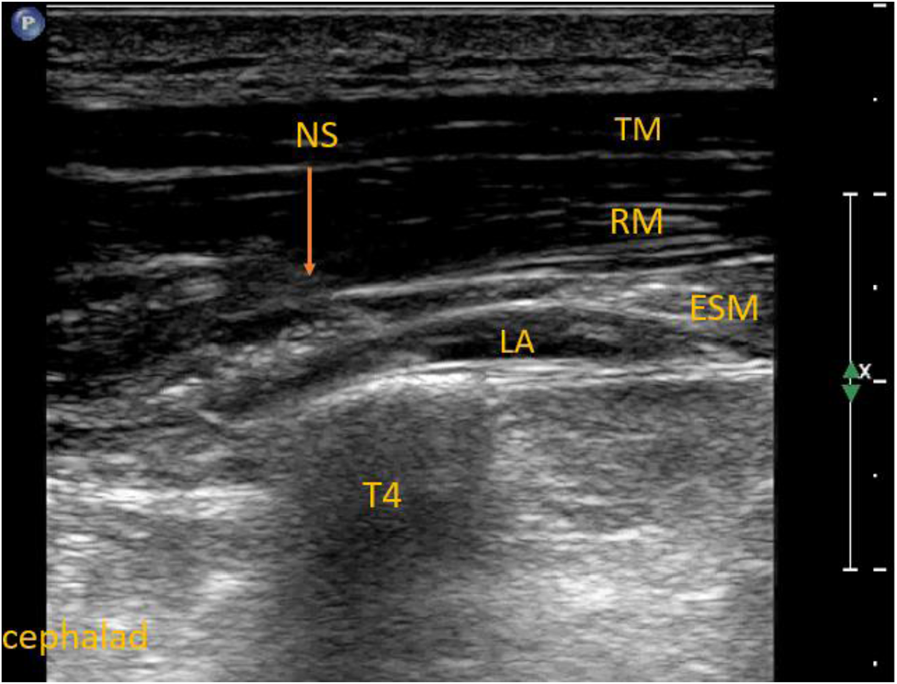
Erector spinae plane block. ESM – erector spinae muscle, LA – local anesthetic, NS- needle shaft, RM- rhomboid muscle, T4 – the transverse process of the fourth thoracic vertebra, TM – trapezius muscle

**Fig. 2:**
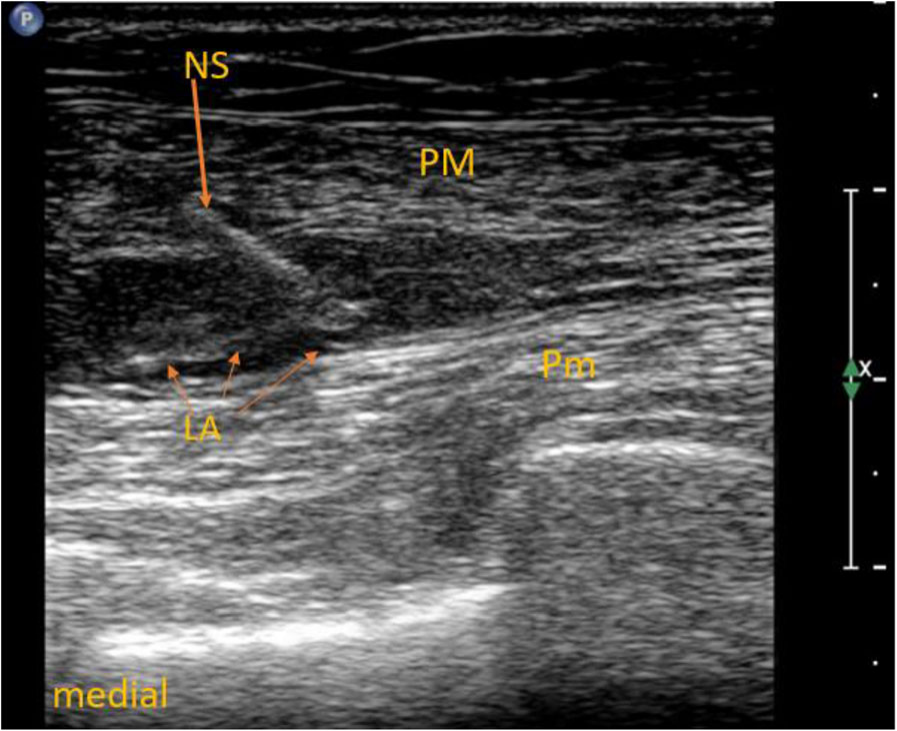
Pectoralis nerves block type I. LA – local anesthetic, NS – needle shaft, PM – pectoralis major muscle, Pm – pectoralis minor muscle

**Fig. 3:**
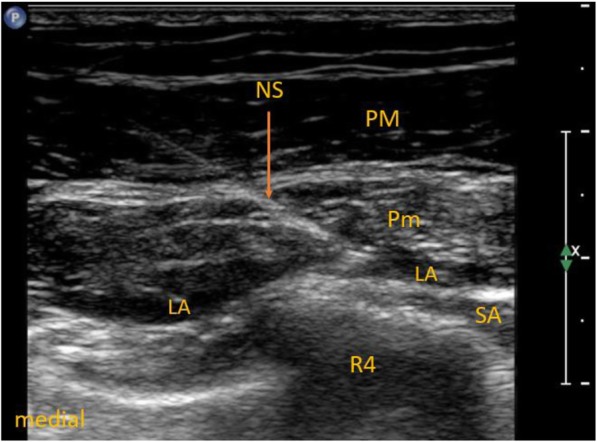
Pectoralis nerves block type II. LA – local anesthetic, NS – needle shaft, PM – pectoralis major muscle, Pm – pectoralis minor muscle, R4 – fourth rib, SA – serratus anterior muscle

In the ESP group, ultrasound-guided ESP block at the fourth thoracic level was performed before the surgery and induction of general anesthesia with Ropivacaine (0.375%; Ropimol, Molteni, Italy, 0.2 mL/kg) as described in our previous study (Fig. 1) [13]. The maximum dosage of ropivacaine could not exceed 20 mL in this group. In the PECS + ESP group, in addition to ESP block, ultrasound-guided PECS blocks type I and II were performed. Local anesthetic (6–8 ml) was deposited in the fascial plane between the pectoralis major and minor muscles (PECS I, Fig. 2); 12–14 ml was deposited between the pectoralis minor and serratus anterior muscles (PECS II, Fig. 3). The total dose of local anesthetic could not exceed 40 mL (150 mg of ropivacaine) in this group.

### Anesthesia

Etomidate (Hypnomidate, Janssen-Cilag International NV, Belgium), remifentanil (0.5–1.0 mcg kg^− 1^ min^− 1^) (Ultiva, GlaxoSmithKline, UK), and rocuronium (0.6 mg kg^− 1^) (Esmeron, N.V. Organon, Holland) were used for the induction of general anesthesia. Maintenance was provided with 0.5 minimum alveolar concentration of sevoflurane (age-adjusted, Sevorane, Abbvie, USA), remifentanil, and incremental doses of rocuronium. Remifentanil was continued to achieve a target plasma concentration of 4–8 ng ml^− 1^ and adjusted to the patient’s heart rate and blood pressure. During the procedure, the right lung was deflated, and the left lung was ventilated with a mixture of air and O_2_. Residual neuromuscular block was reversed with sugammadex (BridionN.V. Organon, Holland) at the end of surgery.

An intravenous bolus of oxycodone (0.1 mg kg^− 1^) was administered 30 min prior to the surgery end. Patients were transferred to the intensive care unit where target plasma concentration of remifentanil was reduced to 0.5–2 ng ml^− 1^. Ventilation was continued for 60–120 min and patients were observed for occurrence of excessive postoperative bleeding and hemodynamic instability. If no problems were recognized, remifentanil infusion was discontinued, and the patient’s trachea was extubated. Postoperative pain treatment was continued with a patient-controlled analgesia (PCA) pump which supplied oxycodone (1 mg per dose, at 7-min intervals, without basal infusion) during the first 24 postoperative hours.

Moreover, intravenous paracetamol, 1 g per 6 h, was administered routinely. Postoperative pain was evaluated by nurses using the VAS at 2, 4, 6, 8, 12, and 24 h postoperatively. Patients could evaluate their pain severity from 0 (no pain) to 100 mm (maximum pain) on the VAS. If pain intensity exceeding 40 mm on the VAS, up to two extra doses of oxycodone (5 mg each, rescue analgesia) could be administered intravenously by the nurse. Patients were transferred to the surgery ward by the end of the first postoperative day if no complications were present.

### Surgery

For mini-invasive mitral and/or tricuspid valve surgery, the patient was placed in the supine position with elevated right hemithorax, and the right upper arm was flexed anteriorly with the forearm in front of the face. Transoesophageal echocardiographic (TEE) monitoring was performed for all patients to confirm the appropriate establishment of cardiopulmonary bypass (CPB), valvular repair, and heart de-airing. The chest was prepared and draped, and the right lung was deflated; a thoracotomy (5 to 7 cm in length) was then performed in the fourth intercostal space in the submammary fold, from the anterior to the medial axillary line. Small accessory incisions were made for the endoscope, aortic clamp, venting tube, CO_2_ line, and atrial retractor.

**Fig. 4:**
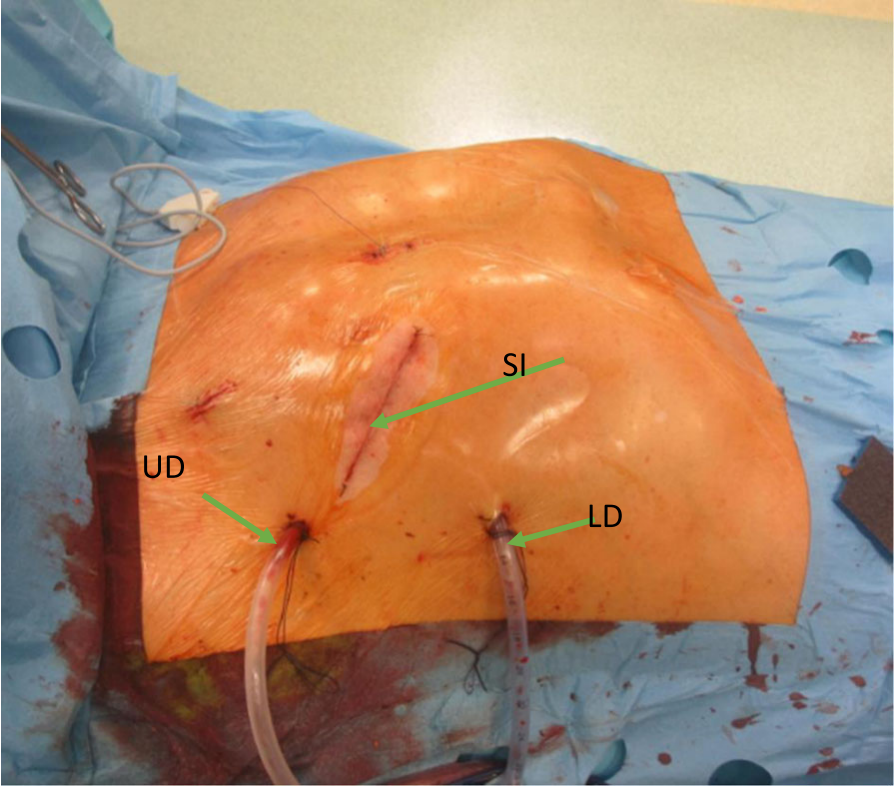
Postoperative drain positions. The figure presents the positions of chest drains and the site of the incision. UD—upper drain, the proximal end in the apex of the lung, LD—lower drain, inserted horizontally (“lying on the diaphragm”), SI—surgical incision

CPB was established via femoral vessel cannulation; if tricuspid valve surgery was also planned, the right jugular vein was cannulated percutaneously. Patients were cooled to 34 °C, the pericardium was opened, and cardioplegia was administered to the aortic root after cross-clamping of the aorta. The mitral and tricuspid valve (if required) was repaired using valvular rings and artificial Gore-Tex chordae, if required. After completion of the repair, patients were rewarmed and weaned from CPB and TEE examination was performed to assure the quality of the repair. The surgery site and the postoperative drain position are presented in Fig. 4.

### Outcomes

#### Primary outcome

The total consumption of oxycodone during the first 24 postoperative hours. This outcome was presented also as morphine equivalence (ME, 1 mg of oxycodone = 1.5 mg of morphine [16]). Secondary outcome: Pain intensity assessed on the VAS at the 2, 4, 6, 8, 12, and 24 h after surgery by nurses who were blinded to the type of treatment.

#### Other outcomes

The other measured variables were pain intensity (assessed by patients using the Prince Henry Hospital Pain Score (PHHPS)), patient satisfaction with pain management, and assessment of pulmonary function. PHHPS was used to assess the effect of analgesia provided by regional block and intravenously administered painkillers on deep breathing and coughing. Patients could describe their pain severity using a five-grade scoring system from 0 to 4, in which 0 indicated ‘no pain on coughing’, 1 indicated ‘pain on coughing, but not on deep breathing’, 2 indicated ‘pain on deep breathing, but not at rest’, 3 indicated ‘slight pain at rest’, and indicated 4 ‘severe pain at rest’. PHHPS was assessed at the time of admission, as well as at 1 day and 4 days after surgery. Patient satisfaction with pain management was assessed at the time of discharge from the hospital. Patients could describe their satisfaction with pain management as perfect (5), good (4), moderate (3), poor (2), or very poor (1).

Pulmonary function tests were performed by a physician who was not involved in anesthesia or surgery. The physician assessed each study participant by using the SP10W spirometer (Contec Medical Systems Co., Ltd., People’s Republic of China) before surgery, as well as 1 day and 4 days after surgery.

### Statistical analysis

Data are presented as medians [interquartile ranges (IQRs)]. The Mann–Whitney U test was used for nonparametric data. If normal distribution was confirmed, Student’s t-test was used. Parametric data are presented as means with 95% confidence intervals (95% CIs). All analyses were performed in Statistica 13.1 software (Stat Soft. Inc., Tulsa, OK, USA).

### Power analysis

The sample size was calculated based on our preliminary results. The mean consumption of oxycodone was 22 mg per day in patients who had the ESP block alone, and 10 mg in patients who had ESP, PECS I, and PECS II blocks. The calculated sample size was 12 individuals per group (α = 0.05; power = 0.8). Thus, we decided to recruit 15 patients in each group.

## Results

**Fig. 5:**
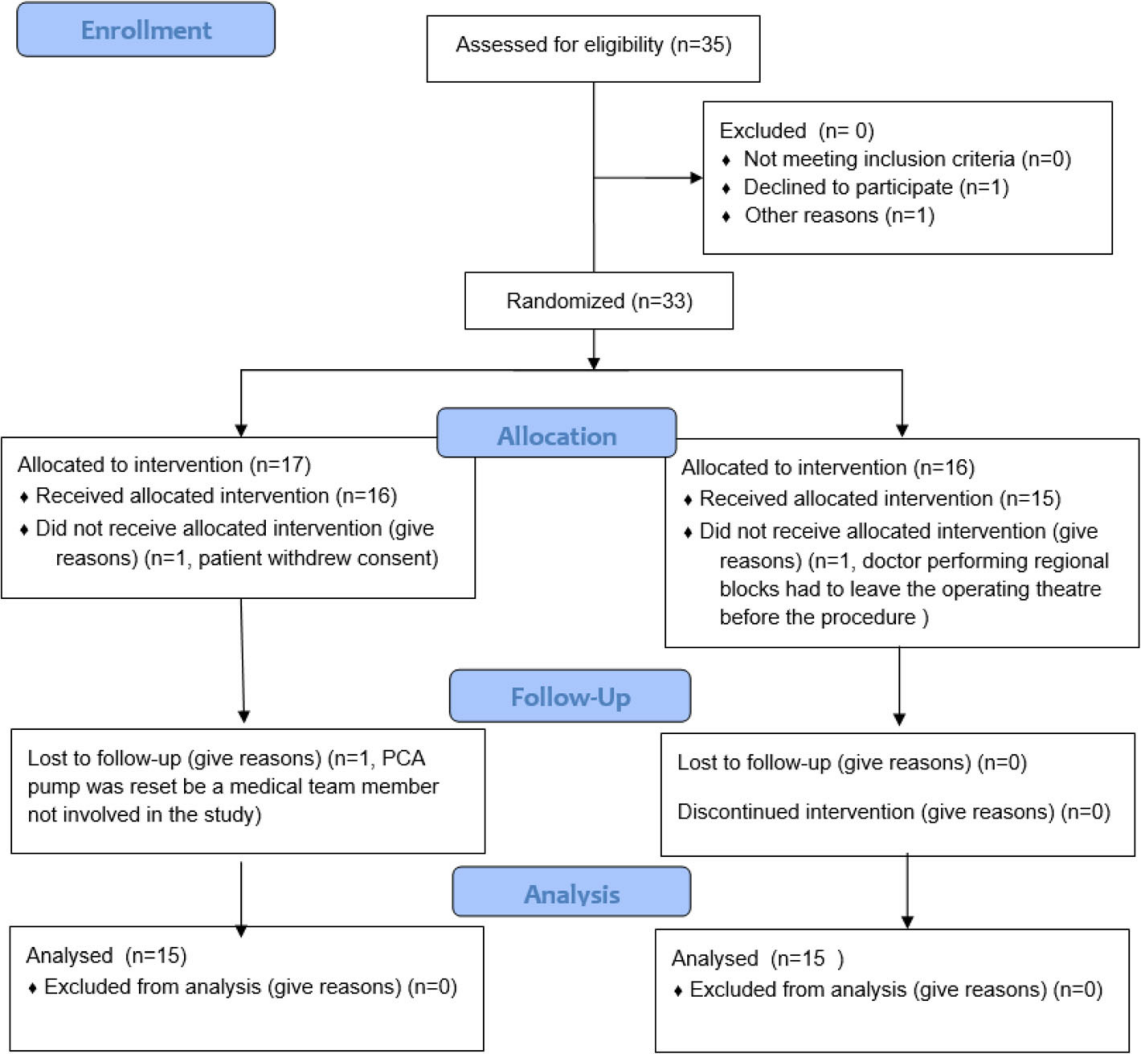
Study flowchart

This study was conducted from July 2018 to August 2018. Overall, 30 patients were analyzed, 15 per group (Fig. 5). Patient demographics and surgery times are presented in Table 1. No differences were found between the groups regarding patient demographics, surgery times, or American Society of Anesthesiologist Physical Status Classifications. We did not notice any relevant complications among the study participants.

**Table 1:** Patient demographics

Group	ESP	PECS + ESP	*p*-value
Age (years)	60. 7 (53.9–67.6)	53.9 (45.7–62.0)	0.18
Weight (kg)	82.3 (75.7–88.9)	79.3 (71.1–87.5)	0.55
Height (m)	175.5 (170.5–180.5)	172.6 (167.1–178.1)	0.41
BMI (kg/m2)	26.9 (24.4–29.4)	26.5 (24.7–28.2)	0.78
Males N (%)	12 (80)	10 (67)	0.68
Surgery time (minutes)	226.7 (207.3–246)	213.7 (189.5–237.8)	0.38
ASA	2 [2–2]	2 [2–2]	0.63

Age, weight, height, body mass index (BMI), and surgery time are shown as means and 95% confidence intervals. American Society of Anesthesiologists Physical Status Classification (ASA) is shown as median and interquartile range. Patient sex is shown as the number (percent) of males in each group. *P*-values were calculated with Student’s t-test (normally distributed continuous data), the Mann–Whitney U test (non-normally distributed data), and the Fisher exact test (frequency data). ESP – erector spinae plane, PECS –pectoralis nerve

### Oxycodone consumption

**Fig. 6:**
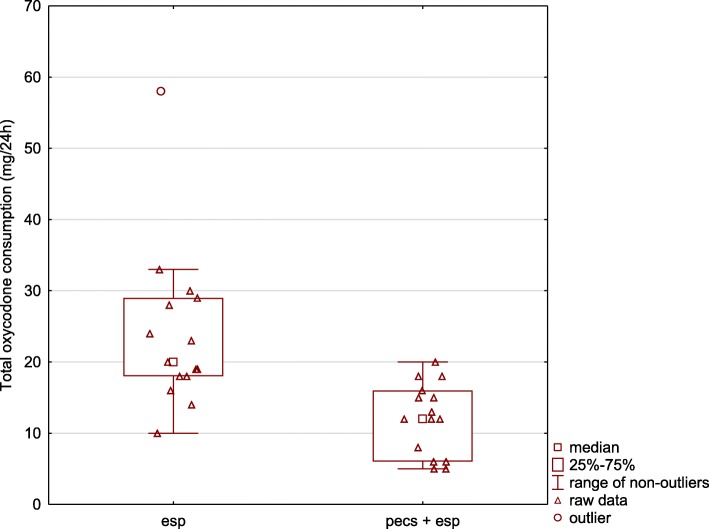
Total oxycodone consumption during the first postoperative day was significantly lower in patients who had PECS I + PECS II + ESP block (PECS + ESP group) than in patients who had ESP block alone. Results are presented as medians and interquartile ranges. ESP – erector spinae plane, PECS – pectoralis nerve

The primary outcome of our study was the oxycodone consumption via PCA during the first 24 postoperative hours. Patients in the PECS + ESP group used significantly less oxycodone than individuals in the ESP group: 12 [IQR: 6–16] mg vs. 20 [IQR: 18–29] mg or 18 [9–24] vs. 30 [27–43.5] ME (*p* = 0.0004) (Fig. 6). Six patients required rescue dosages of oxycodone; all were in the ESP group.

### Pain intensity

**Fig. 7:**
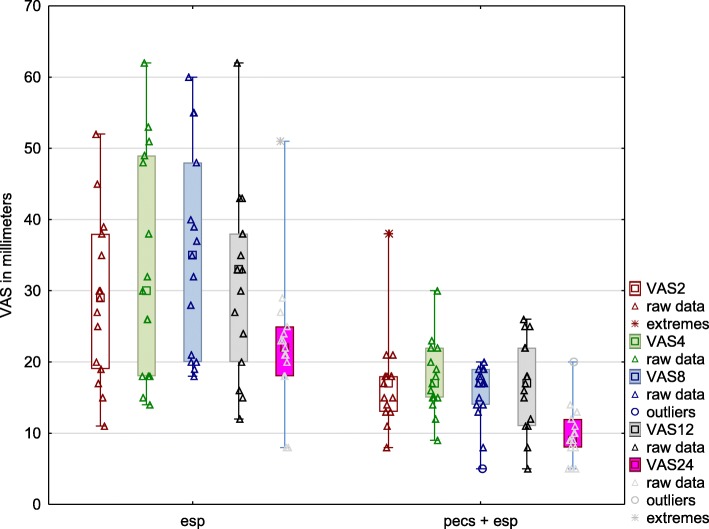
Pain intensities reported by individual patients (triangles) and by groups of patients (boxes and whiskers) using the VAS. Results are presented as medians, 25th–75th percentile ranges (interquartile ranges - boxes), and 1st-99th percentile ranges (whiskers). VAS2, VAS4, VAS8, VAS12, and VAS24 denote pain intensity measurements at the second, fourth, eighth, 12th, and 24th hours postoperatively. ESP – erector spinae plane, PECS – pectoralis nerve, VAS – visual analog scale

Pain intensity was significantly lower in patients in the PECS group, compared with those in the ESP group, at the time of each clinical evaluation (Fig. 7, Table 2).

**Table 2:** Pain intensity

Pain evaluation	ESP	PECS + ESP	*p* value
2 h	29 [19–38]	17 [13–18]	0.004
4 h	30 [18–49]	17 [15–22]	0.005
8 h	35 [18–49]	17 [14–19]	< 0.001
12 h	33 [20–38]	17 [11–22]	0.002
24 h	22 [18–25]	9 [8–12]	< 0.001

Pain intensity reported by patients and presented as medians and interquartile ranges. P-values were calculated with the Mann–Whitney U test. ESP – erector spinae plane, PECS – pectoralis nerve

### Prince Henry hospital pain score

No difference was found between the ESP and PECS + ESP groups regarding pain severity measured on PHHPS. None of the patients reported any pain at the time of admission. In both groups, pain severity was 1 [IQR: 1–1] on the first postoperative day and 1 [IQR: 0–1] on the fourth postoperative day.

### Patient satisfaction with pain management

Patients in the PECS + ESP group were more satisfied with pain management, compared with patients in the ESP group: 4 [IQR: 4–4] vs. 3 [IQR: 1–4] (*p* = 0.0007).

### Pulmonary function tests

Pulmonary function tests did not differ between the study groups for any of the evaluations. Selected parameters from pulmonary function tests are presented in Table 3. Pulmonary function decreased by approximately 30% from baseline but was similar in both groups.

**Table 3:** Pulmonary function tests

Time of assessment	ESP			PECS + ESP		
	FVC (L)	FEV1 (L/s)	PEF (L/s)	FVC (L)	FEV1 (L/s)	PEF (L/s)
Admission	3.3 (2.5–4.0)	2.7 (2.1–3.3)	6.4 (4.9–7.9)	3.4 (3.0–3.8)	2.7 (2.5–3.0)	5.6 (4.8–6.4)
*p*-value	0.73	1.0	0.32			
POD1	2.3 (1.8–2.9)	1.8 (1.4–2.2)	4.7 (3.7–5.6)	2.6 (2.2–3.0)	2.0 (1.7–2.3)	4.8 (3.9–5.7)
*p*-value	0.47	0.35	0.87			
POD4	2.6 (2.1–3.1)	2.1 (1.7–2.5)	6.2 (5.1–7.3)	2.8 (2.4–3.2)	2.2 (1.8–2.6)	5.4 (4.4–6.3)
*p*-value	0.50	0.81	0.21			

Selected results of pulmonary function tests in both groups of patients. Spirometry was performed 1 day before surgery (admission), 1 day after surgery (POD1), and 4 days after surgery (POD4). Data are presented as means and 95% confidence intervals. P-values were calculated with Student’s t-test was. ESP – erector spinae plane, PECS – pectoralis nerve, FVC – forced vital capacity, FEV1 – forced expiratory volume in 1 s, PEF – peak expiratory flow

## Discussion

To our knowledge, this is the first randomized controlled trial (RCT) to compare ESP block with ESP plus PECS I and II blocks in patients undergoing cardiac surgery comprising valve surgery via right mini-thoracotomy. The results of the current study showed that the inclusion of an additional regional anesthesia technique (PECS I + PECS II blocks) with the ESP block significantly reduced oxycodone consumption and alleviated postoperative pain severity measured on the VAS (Figs. 6 and 7). Moreover, patients in the PECS + ESP group were more satisfied with pain management. However, pain management, as measured using the PHHPS, was good in both groups, and there was no difference in pulmonary function tests between the study groups. Of 30 patients, all could be weaned from mechanical ventilation in accordance with the study protocol (within 2 h from the end of the surgery).

ESP block provides satisfactory analgesia in patients after mini-thoracotomy procedures. In the current study, of 15 patients in the ESP group, 12 reported that their pain management was perfect or good; only a single participant reported pain management as poor. However, a continuing obstacle to the improvement of postoperative analgesia remains chest pain associated primarily with chest drains. We considered two regional techniques for additional analgesia: PECS and the serratus anterior block. Both methods have been described in patients who have undergone mini-thoracotomy procedures [15, 17]. We chose to use PECS blocks due to our experience with this method. This modification significantly reduced postoperative pain and improved patient satisfaction in the PECS group.

Both ESP and PECS blocks are relatively new analgesic techniques. ESP is an interfascial plane block developed by Ferrero et al. in 2016 [18]. The deposition of local anesthetic in a location anterior to the erector spinae muscle causes multidermatomal sensory block on the ipsilateral side [19]. PECS blocks require an injection of local anesthetic into two planes: between the pectoralis major and pectoralis minor muscles; and between the pectoralis minor and serratus anterior muscles [15]. These techniques block branches of the brachial plexus (anterior thoracic nerves). Recently, new studies have shown further use of ESP and PECS block in cardiac surgery [11, 12].

Although PECS and ESP blocks appear to cover similar areas, their clinical efficacy is still under investigation. The results presented in cadaveric studies showed some unpredictably of ESP block [19, 20]. In the study by Adhikary et al., the dye spread to the intercostal space was between 5 to 10 spaces, to the epidural space from 2 to 5, and the intercostal foramina from 2 to 3. Thus, the spread of dye in the ESP block was changeable and could differ significantly between only three cadavers. In a very recent study by Choi et al., 14 cadavers were evaluated (7 per group). Two volumes of dye were compared, 10 and 30 mL. Similarly to the previous study, the dye was injected at the level of T5 [20].. Interestingly, the superior costotransverse ligament was stained in 3 of 7 cadavers at the level T3, and only in 1 of 7 cadavers at the T2 level after 30 mL of dye. In the current study, lower pain intensity and better patient satisfaction in the PECS + ESP group could be caused by the covering area not fully supplied by ESP block in some patients. It appears that pain intensity alleviation and improved patient satisfaction could be caused by only PECS II block. PECS I block which covers a small area of the anterior chest wall could be an unnecessary procedure in our trial. However, we cannot fully exclude its usefulness in this case. More evidence is necessary.

Other potential techniques that could be used in patients after mitral and/or tricuspid valve repair via mini-thoracotomy include PVB and TEA. PVB seems superior to TEA for this type of surgery because its analgesic area is limited to the operated side [1, 21]. Data to compare pain relief between ESP and PVB are lacking, but we suspect that their efficacy is similar. However, we hypothesized that PVB could be associated with an increased risk of pleural puncture, relative to that of ESP block [22]. Further RCTs are needed to investigate whether ESP and PVB are equivalent with respect to pain management, complication rate, and patient satisfaction.

The other regional anesthesia method which could be effective after mini-thoracotomy procedures are the intercostal blockade. This procedure could be performed at the end of surgery by the surgeon under direct vision. However, the intercostal blockade provides the highest plasma ropivacaine concentration of all anesthetic techniques, with the peak plasma concentration at 21 ± 9 min from injection and sensory blockade (measured by pinprick) lasting of 6.0 ± 2.5 h only [23].

Our study had some limitations. Although statistical significance was demonstrated for primary and secondary outcomes, the sample size was relatively small. Thus, the lack of complications could be the result of a low number of participants. The current study showed that the addition of PECS block to ESP block improved postoperative pain control and increased patient satisfaction. However, PECS blocks may be sufficient as a single regional analgesia technique for pain management in patients undergoing valve repair via right mini-thoracotomy. Moreover, PECS blocks could be superior to ESP block for this type of surgery. The current study did not exclude this alternative. Neither ESP nor PECS blocks effectiveness was confirmed in the operating theatre with the loss of sensation technique before the surgery.

## Conclusion

In conclusion, the current study demonstrated that the addition of PECS blocks to ESP block led to reduced consumption of oxycodone via PCA, reduced pain intensity on VAS, and increased patient satisfaction with pain management in patients undergoing mitral/tricuspid valve repair via mini-thoracotomy. However, there were no differences between the study groups regarding pulmonary function tests.

## Data Availability

The datasets used and/or analyzed during the current study are available from the corresponding author on reasonable request.

## Abbreviations

CBP: Cardiopulmonary bypass
CI: Confidence interval
ERAS: Enhanced Recovery After Surgery
ESP: Erector spinae plane
IQR: Interquartile range
ME: Morphine equivalence
PCA: Patient-controlled analgesia
PECS: Pectoralis nerve
PHHPS: Prince Henry Hospital Pain Score
PVB: Paravertebral block
TEA: Thoracic epidural analgesia
TEE: Transoesophageal echocardiography
VAS: Visual analog scale
